# 2,3,5-Trimethyl-1,4-hydro­quinone

**DOI:** 10.1107/S1600536810000140

**Published:** 2010-01-09

**Authors:** Jun Dai, Min-Hao Xie, Ya-Ling Liu, Pei Zou, Hao Wu

**Affiliations:** a Jiangsu Institute of Nuclear Medicine, Wuxi 214063, People’s Republic of China

## Abstract

The mol­ecule of the title compound, C_9_H_12_O_2_, is approximately planar (mean atomic deviation = 0.0346 Å) and disposed about a crystallographic centre of symmetry. The H atom of the benzene ring is disordered over four orientations, with occupancies of 0.100 (3) and 0.401 (3) at the C atoms in the 2- and 3-positions and the same at their symmetric location. The H atoms of methyl group at the 2-position are disordered over two positions of equal occupancy. In the crystal structure, adjacent mol­ecules are linked through O—H⋯O hydrogen bonds, forming a two-dimensional network.

## Related literature

The title compound is an important inter­mediate for the preparation of vitamin E, see: Close & Oroshnik (1977 ▶); Mulhauser & Chabardes (1986 ▶); Yao & Han (1999 ▶).
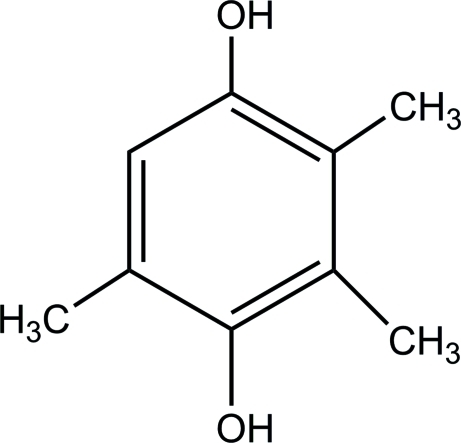


## Experimental

### 

#### Crystal data

C_9_H_12_O_2_
*M* *_r_* = 152.19
Monoclinic, 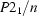

*a* = 8.035 (4) Å
*b* = 4.696 (2) Å
*c* = 10.503 (5) Å
β = 92.813 (5)°
*V* = 395.8 (3) Å^3^
*Z* = 2
Mo *K*α radiation
μ = 0.09 mm^−1^
*T* = 93 K
0.50 × 0.23 × 0.05 mm

#### Data collection

Rigaku SPIDER diffractometer
Absorption correction: ψ scan (North *et al.*, 1968 ▶) *T*_min_ = 0.957, *T*_max_ = 0.996
3724 measured reflections
905 independent reflections
667 reflections with *I* > 2σ(*I*)
*R*_int_ = 0.031
Standard reflections: 0

#### Refinement

*R*[*F* ^2^ > 2σ(*F* ^2^)] = 0.040
*wR*(*F* ^2^) = 0.081
*S* = 1.00
905 reflections
62 parameters
1 restraint
H atoms treated by a mixture of independent and constrained refinement
Δρ_max_ = 0.17 e Å^−3^
Δρ_min_ = −0.11 e Å^−3^

### 

Data collection: *RAPID-AUTO* (Rigaku, 2004 ▶); cell refinement: *RAPID-AUTO*; data reduction: *RAPID-AUTO*; program(s) used to solve structure: *SHELXS97* (Sheldrick, 2008 ▶); program(s) used to refine structure: *SHELXL97* (Sheldrick, 2008 ▶); molecular graphics: *SHELXTL* (Sheldrick, 2008 ▶); software used to prepare material for publication: *SHELXTL*.

## Supplementary Material

Crystal structure: contains datablocks I, global. DOI: 10.1107/S1600536810000140/hg2613sup1.cif

Structure factors: contains datablocks I. DOI: 10.1107/S1600536810000140/hg2613Isup2.hkl

Additional supplementary materials:  crystallographic information; 3D view; checkCIF report

## Figures and Tables

**Table 1:** Hydrogen-bond geometry (Å, °)

*D*—H⋯*A*	*D*—H	H⋯*A*	*D*⋯*A*	*D*—H⋯*A*
O1—H1*O*⋯O1^i^	0.89 (2)	1.92 (2)	2.7833 (14)	164.9 (18)

Symmetry code: (i) 
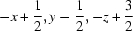
.

**Fig. 1.**
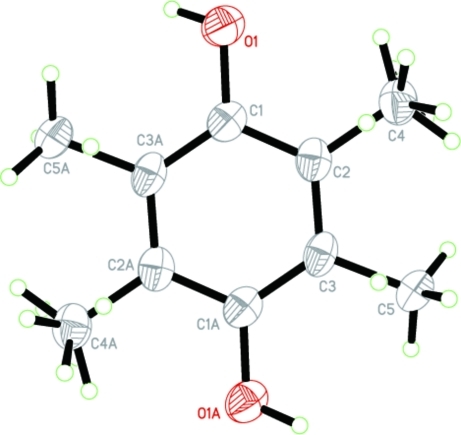
A view of the title compound with the atomic numbering scheme. Displacement ellipsoids were drawn at the 50% probability level.

**Fig. 2.**
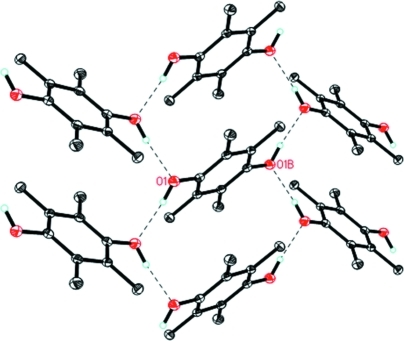
Presentation of the two-dimensional hydrogen-bonded network. Hydrogen bonds are shown as dashed lines.

## supplementary crystallographic information

### Comment

The molecule of the title compound (Fig.1) is useful as an important intermediate for the preparation of vitamin E (Close *et al.*, 1977; Mulhauser *et al.*, 1986; Yao *et al.*, 1999;). We report here the crystal structure of the title compound. The crystal data show that the molecule is approximately planar and and disposed about a crystallographic centre of symmetry. Two hydroxy groups are attached at C1 and C1a of the benzene ring. The only one hydrogen of the benzene ring can be found in other four positions. The occupancies of hydrogen atom(H2') and methyl group(C4) are 0.100 (3) and 0.900 (3) at C2 and the same of its symmetric location(C2a). And the occupancies of H3' and C5 are 0.401 (3) and 0.599 (3) at C3 and C3a. Also the H atoms of methyl group(C4) are disordered over two positions by rotation about its C—C δ bond with equal occupancies.In the crystal structure, adjacent molecules are linked through O—H···O hydrogen bonds to form a two-dimensional hydrogen-bonded network parallel to the [1 0 1] crystallographic plane (Tab 1 and Fig. 2).

### Experimental

A sample of commercial 2,3,5-trimethyl-1,4-hydroquinone(Aldrich) was crystalized by slow evaporation of a solution in benzene: colourless platelet-shaped crystals were formed after several days. ^1^H-NMR (400 MHz; CDCl_3_) δ: 2.145, 2.172, 2.181 (s, 9H, 3×CH_3_), 4.194, 4.213 (s, 2H, 2×OH), 6.453 (s, 1H, Ph—H); ^13^C-NMR(400 MHz; CDCl3) δ: 11.94, 12.28, 15.90(3×*CH_3_*), 114.33 (*Ph*-H), 120.82, 121.02, 123.48 (3×*Ph*-CH_3_), 145.90, 146.94 (2×*Ph*-OH).

### Refinement

The H atom of the benzene ring is disordered over four positions, the occupancies are 0.100 (3),0.401 (3) and the same of their symmetric location. In the case of methyl group(C4), H atoms are disordered over two sites of equal occupancy by rotation about the C—C bonds. The hydroxyl hydrogen was located by difference Fourier synthesis. Other H atoms were placed in geometry calculated positions, taking full account of the disordered noted above, with C—H set to 0.95 Å and 0.98 Å for benzene and methyl H atoms respectively, and refined with a riding model, with *U*_iso_(H) = 1.2*U*_eq_(C) in all cases.

### Crystal data

**Table:**

C_9_H_12_O_2_	*F*(000) = 164
*M**_r_* = 152.19	*D*_x_ = 1.277 Mg m^−^^3^
Monoclinic, *P*2_1_/*n*	Melting point: 442(2) K
Hall symbol: -P 2yn	Mo *K*α radiation, λ = 0.71073 Å
*a* = 8.035 (4) Å	Cell parameters from 930 reflections
*b* = 4.696 (2) Å	θ = 3.1–27.5°
*c* = 10.503 (5) Å	µ = 0.09 mm^−^^1^
β = 92.813 (5)°	*T* = 93 K
*V* = 395.8 (3) Å^3^	Platelet, colorless
*Z* = 2	0.50 × 0.23 × 0.05 mm

### Data collection

**Table:**

Rigaku SPIDER diffractometer	905 independent reflections
Radiation source: Rotating Anode	667 reflections with *I* > 2σ(*I*)
graphite	*R*_int_ = 0.031
ω scans	θ_max_ = 27.5°, θ_min_ = 3.1°
Absorption correction: ψ scan (North *et al.*, 1968)	*h* = −10→10
*T*_min_ = 0.957, *T*_max_ = 0.996	*k* = −6→6
3724 measured reflections	*l* = −13→13

### Refinement

**Table:**

Refinement on *F*^2^	Primary atom site location: structure-invariant direct methods
Least-squares matrix: full	Secondary atom site location: difference Fourier map
*R*[*F*^2^ > 2σ(*F*^2^)] = 0.040	Hydrogen site location: inferred from neighbouring sites
*wR*(*F*^2^) = 0.081	H atoms treated by a mixture of independent and constrained refinement
*S* = 1.00	*w* = 1/[σ^2^(*F*_o_^2^) + (0.0146*P*)^2^ + 0.186*P*] where *P* = (*F*_o_^2^ + 2*F*_c_^2^)/3
905 reflections	(Δ/σ)_max_ < 0.001
62 parameters	Δρ_max_ = 0.17 e Å^−^^3^
1 restraint	Δρ_min_ = −0.11 e Å^−^^3^

### Special details

**Table:**

Geometry. All e.s.d.'s (except the e.s.d. in the dihedral angle between two l.s. planes) are estimated using the full covariance matrix. The cell e.s.d.'s are taken into account individually in the estimation of e.s.d.'s in distances, angles and torsion angles; correlations between e.s.d.'s in cell parameters are only used when they are defined by crystal symmetry. An approximate (isotropic) treatment of cell e.s.d.'s is used for estimating e.s.d.'s involving l.s. planes.
Refinement. Refinement of *F*^2^ against ALL reflections. The weighted *R*-factor *wR* and goodness of fit *S* are based on *F*^2^, conventional *R*-factors *R* are based on *F*, with *F* set to zero for negative *F*^2^. The threshold expression of *F*^2^ > σ(*F*^2^) is used only for calculating *R*-factors(gt) *etc*. and is not relevant to the choice of reflections for refinement. *R*-factors based on *F*^2^ are statistically about twice as large as those based on *F*, and *R*- factors based on ALL data will be even larger.

### Fractional atomic coordinates and isotropic or equivalent isotropic displacement parameters (Å^2^)

**Table:**

	*x*	*y*	*z*	*U*_iso_*/*U*_eq_	Occ. (<1)
O1	0.26338 (14)	0.4290 (2)	0.68009 (10)	0.0409 (3)	
C1	0.38434 (19)	0.4579 (3)	0.58997 (12)	0.0326 (3)	
C2	0.34638 (18)	0.6452 (3)	0.48939 (13)	0.0326 (3)	
C3	0.46482 (19)	0.6852 (3)	0.39874 (12)	0.0325 (3)	
H2'	0.2427	0.7424	0.4831	0.039*	0.100 (3)
H3'	0.4422	0.8107	0.3291	0.039*	0.401 (3)
C4	0.1823 (2)	0.8010 (4)	0.47751 (16)	0.0414 (5)	0.900 (3)
H4A	0.1143	0.7487	0.5489	0.050*	0.4501 (15)
H4B	0.1233	0.7487	0.3970	0.050*	0.4501 (15)
H4C	0.2025	1.0068	0.4790	0.050*	0.4501 (15)
H4D	0.1791	0.9207	0.4010	0.050*	0.4501 (15)
H4E	0.1701	0.9207	0.5529	0.050*	0.4501 (15)
H4F	0.0909	0.6627	0.4709	0.050*	0.4501 (15)
C5	0.4153 (3)	0.8844 (5)	0.2919 (2)	0.0353 (7)	0.599 (3)
H5A	0.2957	0.8652	0.2707	0.042*	0.599 (3)
H5B	0.4779	0.8375	0.2169	0.042*	0.599 (3)
H5C	0.4400	1.0808	0.3182	0.042*	0.599 (3)
H1O	0.273 (2)	0.263 (5)	0.7202 (18)	0.072 (7)*	

### Atomic displacement parameters (Å^2^)

**Table:**

	*U*^11^	*U*^22^	*U*^33^	*U*^12^	*U*^13^	*U*^23^
O1	0.0545 (7)	0.0301 (6)	0.0389 (6)	0.0038 (5)	0.0109 (5)	0.0004 (5)
C1	0.0468 (9)	0.0222 (7)	0.0290 (7)	−0.0009 (6)	0.0030 (6)	−0.0044 (5)
C2	0.0429 (8)	0.0217 (7)	0.0326 (7)	0.0022 (6)	−0.0034 (6)	−0.0049 (5)
C3	0.0493 (9)	0.0201 (7)	0.0276 (7)	0.0017 (6)	−0.0033 (6)	−0.0012 (5)
C4	0.0475 (11)	0.0346 (9)	0.0417 (9)	0.0054 (8)	−0.0014 (8)	0.0013 (7)
C5	0.0434 (14)	0.0308 (13)	0.0314 (12)	−0.0034 (11)	−0.0004 (10)	0.0051 (10)

### Geometric parameters (Å, °)

**Table:**

O1—C1	1.3961 (17)	C4—H4A	0.9800
O1—H1O	0.89 (2)	C4—H4B	0.9800
C1—C3^i^	1.386 (2)	C4—H4C	0.9800
C1—C2	1.3968 (19)	C4—H4D	0.9800
C2—C3	1.392 (2)	C4—H4E	0.9800
C2—C4	1.508 (2)	C4—H4F	0.9800
C2—H2'	0.9498	C5—H3'	0.5575
C3—C1^i^	1.386 (2)	C5—H5A	0.9800
C3—C5	1.500 (3)	C5—H5B	0.9800
C3—H3'	0.9500	C5—H5C	0.9800
C4—H2'	0.5584		
			
C1—O1—H1O	111.0 (12)	H4A—C4—H4C	109.5
C3^i^—C1—O1	122.01 (13)	H4B—C4—H4C	109.5
C3^i^—C1—C2	121.85 (13)	C2—C4—H4D	109.5
O1—C1—C2	116.13 (13)	H2'—C4—H4D	110.8
C3—C2—C1	118.12 (13)	H4A—C4—H4D	141.1
C3—C2—C4	120.17 (13)	H4B—C4—H4D	56.3
C1—C2—C4	121.72 (14)	H4C—C4—H4D	56.3
C3—C2—H2'	121.0	C2—C4—H4E	109.5
C1—C2—H2'	120.9	H2'—C4—H4E	108.6
C4—C2—H2'	0.8	H4A—C4—H4E	56.3
C1^i^—C3—C2	120.04 (12)	H4B—C4—H4E	141.1
C1^i^—C3—C5	124.41 (15)	H4C—C4—H4E	56.3
C2—C3—C5	115.54 (15)	H4D—C4—H4E	109.5
C1^i^—C3—H3'	120.0	C2—C4—H4F	109.5
C2—C3—H3'	120.0	H2'—C4—H4F	109.0
C5—C3—H3'	4.5	H4A—C4—H4F	56.3
C2—C4—H2'	1.4	H4B—C4—H4F	56.3
C2—C4—H4A	109.5	H4C—C4—H4F	141.1
H2'—C4—H4A	108.1	H4D—C4—H4F	109.5
C2—C4—H4B	109.5	H4E—C4—H4F	109.5
H2'—C4—H4B	110.3	C3—C5—H3'	7.7
H4A—C4—H4B	109.5	H3'—C5—H5A	116.4
C2—C4—H4C	109.5	H3'—C5—H5B	103.1
H2'—C4—H4C	109.9	H3'—C5—H5C	108.7
			
C3^i^—C1—C2—C3	0.3 (2)	C1—C2—C3—C1^i^	−0.3 (2)
O1—C1—C2—C3	178.66 (12)	C4—C2—C3—C1^i^	179.88 (13)
C3^i^—C1—C2—C4	−179.88 (13)	C1—C2—C3—C5	178.88 (14)
O1—C1—C2—C4	−1.48 (19)	C4—C2—C3—C5	−1.0 (2)

Symmetry codes: (i) −*x*+1, −*y*+1, −*z*+1.

### Hydrogen-bond geometry (Å, °)

**Table:**

*D*—H···*A*	*D*—H	H···*A*	*D*···*A*	*D*—H···*A*
O1—H1O···O1^ii^	0.89 (2)	1.92 (2)	2.7833 (14)	164.9 (18)

Symmetry codes: (ii) −*x*+1/2, *y*−1/2, −*z*+3/2.
